# Surface Modification by Media Organics Reduces the Bacterio-toxicity of Cupric Oxide Nanoparticle against *Escherichia coli*

**DOI:** 10.1038/s41598-019-51906-2

**Published:** 2019-10-25

**Authors:** Ruchira Chakraborty, Tarakdas Basu

**Affiliations:** Department of Biochemistry and Biophysics, University of Kalyani, Kalyani, 741235 West Bengal India

**Keywords:** Biotechnology, Materials science

## Abstract

Prevalence of antibiotic-resistant bacteria demands alternatives to antibiotics. Copper-based nanoparticles with a high antibacterial property may be a solution to the problem. It is, therefore, important to understand the mode of antibacterial action of the nanoparticles (NPs). Despite reports on induction of reactive oxygen species (ROS) in bacteria by copper and copper-oxide nanoparticles and involvement of such ROS in cell killing, it is still unclear (a) if surface modification of the nanoparticles by media organics has any role on their antibacterial potency and (b) whether the bactericidal effects of these NPs are ‘particle-specific’ or ‘ion-specific’ in nature. We address these issues for cupric oxide nanoparticle (CuO-NP) in this study. Instead of nutrient medium, when *E. coli* bacterial cells were suspended in saline (0.9% NaCl), CuO-NP had a more anti-bacterial effect, with MBC (minimum bactericidal concentration) value of 6 µg/mL, than in nutrient medium with MBC value of 160 µg/mL. Moreover, the lysine-modified CuO-NP in saline had MBC at 130 µg/mL. Thus, unmodified CuO-NP was more efficient killer than modified one. Our finding further revealed that in saline;CuO-NP had ‘particle-specific’ antibacterial effect through generation of ROS and consequent oxidative damage by lipid peroxidation, protein oxidation and DNA degradation in cells.

## Introduction

The rapid increase of different antibiotic-resistant bacterial strains is leading to a critical threat towards the entire mankind in near future^1^. The abandon and inappropriate use of antibiotics ascribe to anti-microbial resistance against an array of different classes of antibiotics. All antibiotics work in a site-specific manner, through either disruption or inhibition of cell wall synthesis, impairment of transcription or translation process, or hampering the nucleic acid synthesis. However, within few years of arrival of each new antibiotic in the market, bacteria are found to evolve new resistance to deal with the antibiotic threat^2–4^. To control the situation, the World Health Organization (WHO) has published a list of antibiotic resistant pathogens, against which development of new anti-microbial agents is of immediate need. In search of alternatives to antibiotics, with high antibacterial activity, metal-based nanoparticles have shown the most promising potency to be used as new antibacterial agents in future^3,5,6^. Most importantly, by the virtue of induction of ROS, metal-based NPs can target multiple biomolecules at once and thus avoid the chance of development of new resistant strains.

Copper and its complexes are popular for their antibacterial action, even from the time before the invention of penicillin. However, their doses, required to eliminate the bacterial infection, are high enough to cause concomitant damage to nearby healthy cells too. Therefore, the direct use of copper complexes is forbidden for the treatment of bacterial infections^7,8^. Recent studies have established high antibacterial potency of copper-based NPs within a considerable low dose range^9–11^. It implies that copper-based NPs can be used as a potential alternative to antibiotics. Before going to use these NPs in the treatment of bacterial infection, a detailed investigation is necessary to unveil the mechanism behind their antibacterial action. Some groups had already attempted to explore the pathway of baterio-toxication of metallic copper, cupric oxide and cuprous oxide NPs^12–21^. The results unraveled that (a) the NPs induced reactive oxygen species (ROS) like superoxide anion (O_2_^• −^), hydroxyl radical (^•^OH), singlet oxygen (^1^O_2_) etc. in bacterial cells and (b) the ROS finally killed cells by causing lipid peroxidation, protein oxidation, chromosomal DNA degradation, and membrane depolarization. However, reports on the mode of ROS induction in cells left an open debate. Few groups have reported that Cu^+^ ^2^ ions, leached from the NPs, were ultimately responsible for antibacterial action i.e., the bacterio-toxic activity of the NPs was an ‘ion-specific’ effect^16–18^; on the contrary others reported that the NPs, as a whole, had antibacterial action as ‘particle specific’ effect^19–21^.

In a previous article, we reported about the preparation of a rare suspended colloidal form of CuO-NPand about its biological and chemical mechanisms of antibacterial action^20^. We found that the bacterial growth media organics interacted and altered the surface of CuO-NPs. Such modified NPs caused induction of ROS and consequent killing of bacterial cells^20^. This left us with the query that if the modification was the sole reason for the antibacterial effect of CuO-NP. In this communication we report that the unmodified CuO-NPs also had antibacterial effect i.e., the NPs could kill *E. coli K-12* cells, suspended in saline, where there was no media organics for modifying CuO-NP. Notably, the MBC of unmodified CuO-NP was significantly lower than the modified one. Therefore, the modification phenomenon actually reduced particle toxicity. Moreover, the unmodified NP was found to have ‘particle specific’ antibacterial effect through induction of intracellular ROS and ROS-mediated damage of cellular lipids, proteins, and DNA.

## Results and Discussion

In our previous communication, we reported the synthesis of a rare suspended colloidal form of CuO-NP at physiological pH 7.5. In the method of synthesis, CuCl_2_ (as a precursor) was first reduced by sodium borohydride (NaBH_4_) in presence of polyvinyl alcohol (PVA, as a stabilizing agent) and then left for simple aerial oxidation to have a leafy green colored suspension of CuO-NPs. The particles were about 50 nm in size, cubic in shape, crystalline in nature with crystal planes (111), (111) and (002) and semi-conductive in nature having two bandgap energies of 3.40 and 3.96 ev corresponding to two characteristic absorption maxima at 266 and 370 nm respectively. The NPs remained stable for 2–3 months under ambient condition. The MBC values of CuO-NP against *E. coli K-12* and *S. aureus* were about 160 and 195 µg/mL respectively, in growth medium Luria-broth (LB). We also found that with the addition of CuO-NPs to LB, the particles were modified by the components of LB, leading to an increase in particle size from around 50 to 230 nm. The modified NPs caused cell death by enhancing the intracellular level of ROS, which subsequently led to oxidative damage of cellular lipid, protein, and DNA. In the present study, our prime objectives were to investigate the antibacterial potency as well as mechanism of unmodified CuO-NP. Here, the experiments were carried out on *E. coli K12* cells by suspending them in normal saline (0.9% NaCl) for treatment with the NP.

### Antibacterial potency of CuO-NP on *E. coli K12* cells, suspended in saline

Freshly grown *E. coli K12* (*wild type*) cells (not resistant to any antibiotic) in LB medium were synchronized in the starvation buffer (SB), as described in the Methods section and were then transferred in saline (0.9% NaCl) to treat/incubate with different concentrations of CuO-NP for 18 h. The viability of cells was determined from colony counts on agar plates. The result depicted that in salines, the MBC value of CuO-NP (which killed cells by three orders or by 99.9%) was around 6.0 µg/mL and 100% cell killing occurred at around 9.0 µg/mL (Table 1). As a control experiment, when the cells were treated with 6.0 µg/mL CuCl_2_ (the prime precursor of the NP), the cell viability decreased by only about one order or by 90% [result not shown]. It implies that CuO-NP had more efficient antibacterial activity than the free ionic precursor CuCl_2_. Here, it should be mentioned that the MBC value of our CuO-NP in LB nutrient medium was 160 µg/mL^20^, which was much higher than that in saline (6 µg/mL) i.e., in saline, where there was no modification of the NP by media organics (as was happened in LB medium)^20^, CuO-NP showed pronounced bacterio-toxicity. Applerot *et al*.^19^ and Pandey *et al*.^21^ separately reported that in saline, the MBC of CuO-NP for *E. Coli* was about100 and 10 µg/mL respectively, which implied that our CuO-NP had higher antibacterial potency (with less MBC value of 6 µg/mL), compared to the referred CuO-NPs. It should be noted here that as Applerot *et al*.^19^ and Pandey *et al*.^21^ incubated the cells with their CuO-NPs for 3 and 2 h respectively (much less than our 18 h of incubation) to obtain the MBC values, we also studied the time-dependent bactericidal effect of our CuO-NP (Supplementary Table S1) in saline. The result indicated that incubation of cells with our CuO-NP (6 µg/mL) for 6 h was sufficient to kill the cells by three order; however, within 6–18 h, CuO-NP treatment reduced the cell population only in numbers, not in orders. Since the standard way to report MBC is after 18–24 h of treatment^9,10^, we preferred to treat the bacterial cells with the NPs for 18 h to obtain the MBC value.

**Table 1:** Number of viable *E. coli K-12* cells after 18 h of incubation in saline with varying concentrations of CuO-NP/lysine-modified CuO-NP. Results are representatives of three independent experiments. Data represent mean values ± S.D.

Concentration of CuO-NP	Number of viable *E. coli K-12* cells	Concentration of lysine-modified CuO-NP	Number of viable *E. coli K-12* cells
0.0 µg/ml	2.0 ± 0.15 × 10^8^	0.0 µg/ml	2.0 ± 0.32 × 10^8^
1.5 µg/ml	6.3 ± 0.19 × 10^7^	15.0 µg/ml	6.2 ± 0. 45 × 10^7^
3.0 µg/ml	1.2 ± 0.38 × 10^7^	60.0 µg/ml	7.5 ± 0.26 × 10^6^
4.5 µg/ml	5.7 ± 0.23 × 10^6^	100.0 µg/ml	1.2 ± 0.52 × 10^6^
**6.0 µg/ml**	**3.1 ± 0.16 × 10** ^**5**^	**130.0 µg/ml**	**5.3 ± 0.38 × 10** ^**5**^
7.5 µg/ml	1.6 ± 0.41 × 10^4^	160.0 µg/ml	7.6 ± 0.46 × 10^3^
9.0 µg/ml	0 × 10	180.0 µg/ml	0 × 10

The result of our study on bactericidal efficiency of CuO-NPs against Gram-positive *Staphylococcus aureus* (*wild type*) cells (not resistant to any antibiotic) depicted that in saline the MBC of CuO-NP for *S. aureus* cells were 7.5 µg/ml (Supplementary Table S2), while in LB the MBC was 195 µg/mL^20^. Therefore, so far as the anti-bacterial potency of CuO-NP is concerned, both the modified (in LB) and unmodified (in saline) CuO-NP have antibacterial potency against both Gram-negative *E. coli* and Gram-positive *S. aureus*; the unmodified one has far effective bacteriotoxicity than the modified one and the *E. coli* is less susceptible to the NP than *S. aureus* (perhaps due to thinner peptidoglycan cell wall of the Gram-negative bacteria than the Gram-positive ones). However, a point may be raised here that the unmodified CuO-NPs acted on metabolically inactive cells in saline and the modified CuO-NPs acted on metabolically active cells in the nutrient medium and so, the difference between the MBC values in saline and nutrient medium might be due to the disparity of cellular conditions (metabolically inactive and active, respectively), but not because of higher antibacterial activity of unmodified NPs over modified ones. In order to resolve this point, cells were treated with varying concentrations of lysine-modified CuO-NP in saline (which was prepared by mixing 500 µg lysine in 360 µg/ml NP suspension, as described in our previous report^20^) with a view that if the metabolic inactivity of cell was the reason of pronounced cytotoxicity of the NPs, then both the lysine-modified and unmodified NPs would show nearly the same range of MBC value. The justification behind assuming the lysine-modified CuO-NPs as similar to the modified CuO-NPs in nutrient medium was that (a) the interaction pattern of CuO-NPs with either of the amino acids Lys, Glu, Ser and Gly was individually similar (shown in Supplementary Fig. S1) and also similar to the interaction pattern of CuO-NPs with the mixture of 20 amino acids (shown in our earlier report^20^) and (b) the pattern of absorption spectra due to stepwise addition of pre-formed NP-Lys complex in LB medium, with absorption maxima at 616 nm (shown in Supplementary Fig. S2) was similar to the pattern due to stepwise addition of unmodified CuO-NP in LB (shown in reference^20^). In saline, the MBC of lysine-modifiedNPs was found to be 130 µg/ml (Table 1), which was) close to the MBC of modified CuO-NPs in the nutrient medium (160 µg/ml)^20^ and b) much higher than the MBC of the unmodified-NP (6 µg/mL) in saline. Table 1 also shows that in saline, the lysine-modified NPs at a concentration of 180.0 µg/mL caused 100% cell killing, whereas the unmodified CuO-NPs at a concentration of only 9.0 µg/mL caused the same. So, the above results signified that the modification of CuO-NPs by media organics reduced the bactericidal activity of the NPs and the unmodified particles had pronounced cytotoxicity in saline, clearly because of their high antibacterial potency, but not for the metabolic inactiveness of the cells in saline.

**Figure 1:**
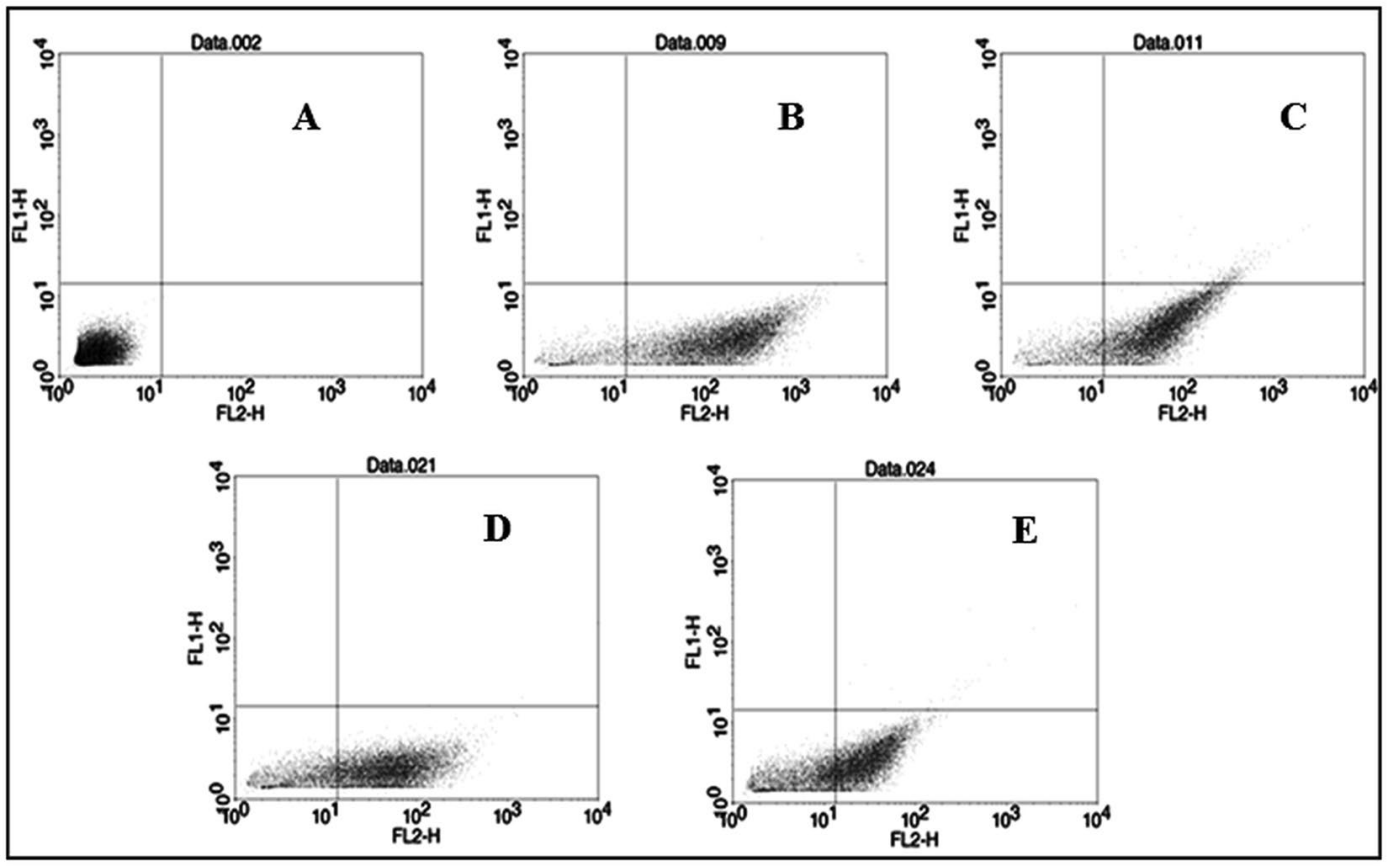
Flow cytometric analysis of membrane potential of *E. coli* cells in saline. (**A**) Untreated control cells without the dye (3,3′-diphenylthiocarbocyanine iodide) labeling. (**B**) Untreated control cells labeled with the dye. (**C**) Cells treated with 6 μg/mL CuCl_2_ and labeled with dye. (**D**) Cells treated with 3 μg/mL CuO-NP and labeled with dye and (**E**) Cells treated with 6 μg/mL CuO-NP and labeled with dye. This experiment was repeated thrice and the result of one has been represented here.

Depolarization of membrane potential was known to be associated with cell death^13,22^. We, therefore, investigated the role of CuO-NP on the membrane potential of *E. coli* cell kept in saline. The result of flow cytometric experiment, as described in the method section, is presented in Fig. 1 and Table 2. The three main quadrants LR and UR (lower right and upper right respectively) and LL (lower left) of the dot plot (Fig. 1) represented the effect of CuO-NP on the membrane potential of cells, labeled with a fluorescent dye (3,3′-diphenylthiocarbocyanine iodide). The dots in the LL quadrant represented the cells, which were not labeled and the dots in the LR and UR quadrants represented labeled fluorescent cells. The dye readily incorporates in cells with a polarized membrane. The result of our control experiment on CuO-NP-untreated cells, after labeling with the dye for 2 h, depicted that about 90% cells became fluorescent (dots at LR and UR in Fig. 1B and Table 2) and only about 10% remained unlabeled (dots in LL in Fig. 1B and Table 2). This implied that 10% of cells in a normal population existed with depolarized membrane. For cells treated with CuO-NP a concentration of 3 µg/mL (sub-MBC, Fig. 1D) and 6 µg/mL (MBC, Fig. 1E), about 82 and 64.5% labeled cells respectively were found in (LR + UR) quadrants and the remaining 18 and 35.5%un-labeled cells were found in LL quadrant. Hence, CuO-NP treatment caused depolarization of membrane potential in a dose depended manner. On the other hand, for the cells treated with 6 µg/mL of precursor CuCl_2_, an insignificant depolarization was observed (Fig. 1C) with 88.75% labeled cells and 11.25% un-labeled cells. Since the membrane depolarization is known to cause cellular death, therefore the increase of membrane depolarization with an increase of NP concentration clearly indicated that CuO-NP had a dose-dependent bactericidal effect.

**Table 2:** Percent distribution of cells in different quadrants of flow cytometric dot-plots.

Sample	Lower Left (LL)	Lower Right (LR)	Upper Right (UR)
Unlabelled control cells	**99.96%**	**0.01%**	**0.02%**
Labelled control cells	**9.96%**	**89.25%**	**0.79%**
3 µg/mL CuO-NP treated cells	**17.95%**	**81.91%**	**0.14%**
6 µg/mL CuO-NP treated cells	**35.37%**	**64.05%**	**0.58%**
6 µg/mL CuCl_2_ treated cells	**11.26%**	**85.75%**	**2.99%**

### Generation of cellular ROS by CuO-NP in saline

Generation of oxidative stress in bacterial cells by treatment with metal-based NPs are manifested by an enhanced level of cellular ROS viz., superoxide anion (O_2_^• −^), hydroxyl radical (^•^OH), singlet oxygen (^1^O_2_), etc., which cause cellular lipid peroxidation, protein oxidation and DNA degradation, finally killing cells^23^. Our earlier study on the mechanism of antibacterial activity of CuO-NP showed that the NP got modified by organics of the cell growth medium LB and the modified CuO-NPs caused induction of cellular ROS with consequent damage in important bio-molecules like lipid and DNA of bacterial cells^20^. Here also, studies were performed to investigate the effect of CuO-NP treatment on cellular induction of ROS in saline and its consequence on lipid peroxidation, protein oxidation, and chromosomal DNA degradation in *E. coli K12* cells.

**Figure 2:**
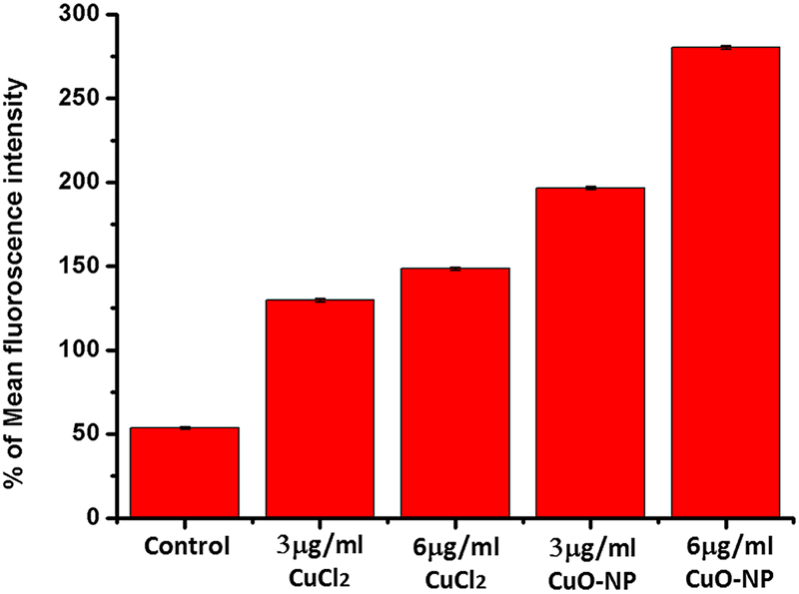
Measurement of cellular ROS in CuO-NP treated *E. coli K-12* cells in saline. Error bar represents mean values ± S.D. (standard deviation) from three independent experiments.

CuO-NP treatment enhanced the cellular ROS level in saline (Fig. 2). At the concentrations of 3 (sub-MBC) and 6 µg/mL (MBC) of CuO-NPs, ROS level in *E. coli* K12 cells was about 3.7 and 5.2 folds respectively of that in untreated control cells (Fig. 2). On the other hand, in cells treated with 3 and 6 µg/mL of CuCl_2_ (NP precursor), the cellular ROS level was about 2.7 and 2.8 folds respectively of the control cells. This result qualitatively supported the findings of Applerot *et al*.^19^, where 8 folds increase of ROS level was reported.

**Figure 3:**
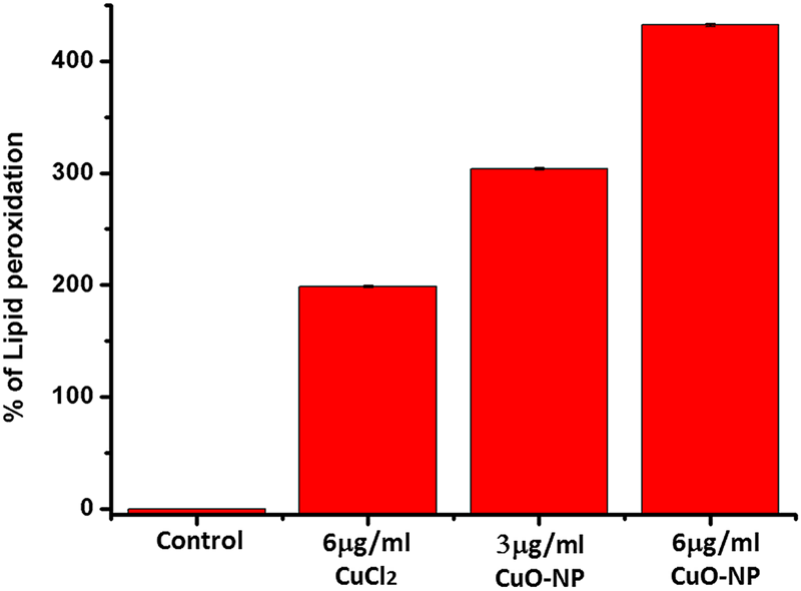
Measurement of cellular LPO in CuO-NP treated *E. coli K-12* cells in saline. Error bar represents mean values ± S.D. (standard deviation) from three independent experiments.

The increased ROS level caused lipid peroxidation in cells. Here, treatment of the saline-starved cells with 3 and 6 µg/mL of CuO-NPs increased the lipid peroxidation level by about 300 and 430% respectively, over the level in untreated control cells. However, treatment with 6 µg/mL of precursor CuCl_2_ increased the level by only about 200% over the control cells’ level (Fig. 3).

**Figure 4:**
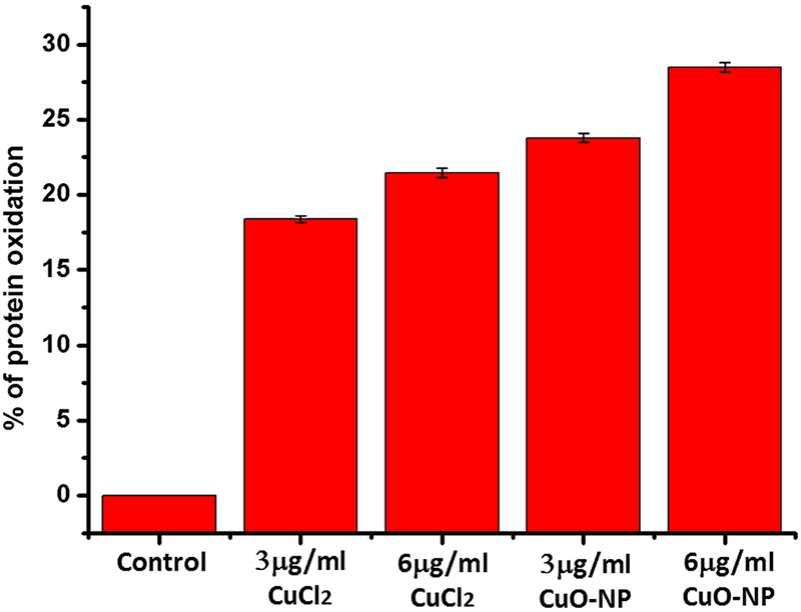
Measurement of cellular protein oxidation in CuO-NP treated *E. coli K-12* cells in saline. Error bar represents mean values ± S.D. (standard deviation) from three independent experiments.

Figure 4 exhibits the effect of CuO-NP/precursor CuCl_2_ on cellular protein oxidation in saline. The treatment with 3 and 6 µg/mL of CuO-NPs or CuCl_2_ increased cellular protein oxidation level by23.8 and 28.5% or 18.4 and 21.5% respectively over the levels in untreated control cells. No protein oxidation was found to occur in growing cells in LB medium by the action of CuO-NPs at MBC (160 µg/mL)^20^; however, protein oxidation occurred in non-growing cells in saline by the NP at MBC (6 µg/mL). This result further implied that CuO-NP was more potent to cause an antibacterial effect on metabolically inactive cells than on metabolically active ones.

**Figure 5:**
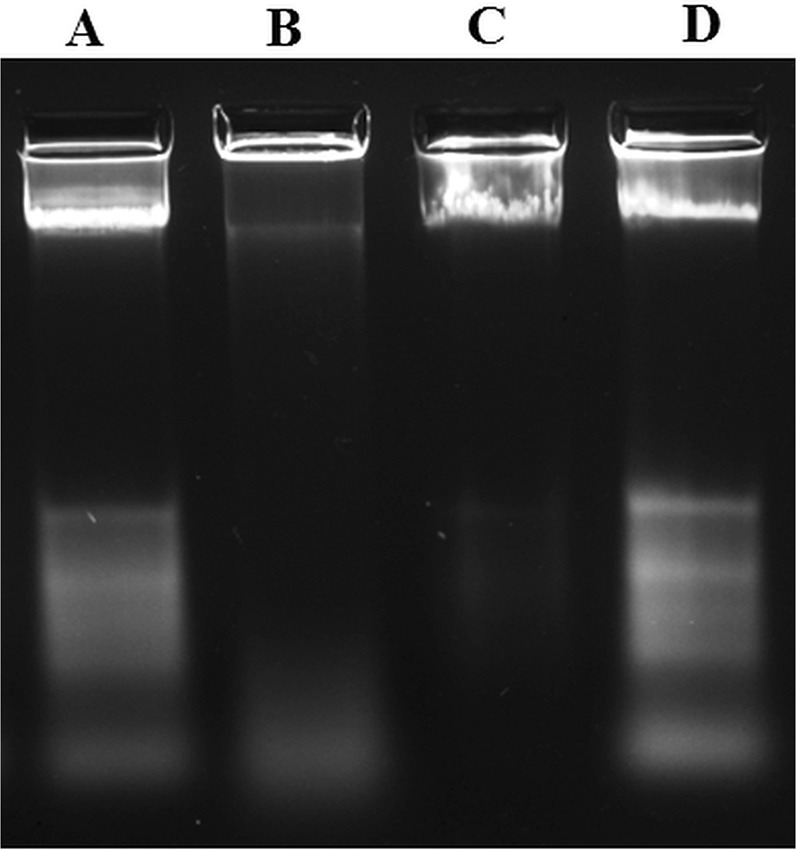
Genomic DNA degradation pattern in CuO-NP treated *E. coli K-12* cells in saline. Lane A: untreated control cells, Lane B: cells treated with 3 μg/mL CuO-NP, Lane C: cells treated with 6 μg/mL CuO-NP and Lane D: cells treated with 6 μg/mL CuCl_2_.

The genomic DNA degradation pattern of CuO-NP-treated cells in saline is shown in Fig. 5. Here, lanes A, B, C, and D represented genomic DNA profile of untreated control cells, 3 µg/mL CuO-NP treated cells, 6 µg/mL CuO-NP treated cells and 6 µg/mL CuCl_2_ treated cells, respectively. The almost same intensity and bands pattern of DNA for untreated control (lane A) and CuCl_2_ treated (lane D) cells indicated that CuCl_2_ had no DNA degrading role. However, a clear DNA degradation pattern was evident from the low intense profile of DNA, isolated from CuO-NP treated cells. The decrease of intensity indicates degradation of DNA into small fragments, that went out of the gel during electrophoresis. The degradation was more prominent in 6 µg/mL CuO-NP treated cells (lane C) than in 3 µg/mL CuO-NP treated ones (lane B). Therefore, this result again demonstrated clearly the dose-dependent cytotoxicity of CuO-NPs in saline.

### The antibacterial efficacy of CuO-NP in saline in the presence of divalent metal ion chelators

The bacterio-toxicity assay of CuO-NP on *E. coli K12* cells, suspended in saline, was also carried out in presence of the divalent metal ion chelators - EDTA (ethylene di-amine tetraacetate) and EDA (ethylene di-amine). The structural difference between EDTA and EDA is that EDA possesses free amine groups, which are altered to acetates in EDTA. As our CuO-NP was shown to interact readily with free amines and got modified, it was therefore expected that the NP might behave differently in the presence of EDTA and EDA. However, both EDTA and EDA being ion chelator are toxic to the cells. Therefore, the viability of cells in saline in the presence of varying concentrations of EDTA and EDA were first determined (Supplementary Table S3). The concentration of EDTA or EDA for subsequent studies was standardized as 2 mM, the presence of which in saline killed cells by less than one order (from 2.2 × 10^8^ to about 5.0 × 10^7^ cells/mL, Table 3). Table 3 also depicts that the extent of cell killing (by about 3-order) due to treatment with CuO-NP at its MBC for 18 h was not altered by the presence or absence of 2 mM EDTA; even treatment of the cells with CuO-NPs pre-incubated with EDTA did not alter the MBC of CuO-NP. These results clearly indicated that EDTA had no effect on the antibacterial potency of the NP, which further implied that the antibacterial effect of CuO-NP was not an ion-specific effect. This proposition was supported by the fact that the antimicrobial activity of CuO-NPs (6 µg/mL) in saline was 100 times higher than that of its precursor CuCl_2_ (6 µg/mL), though in the later environment copper was present in ionic form. On the other hand, cell-killing efficacy of 2 mM EDA (from 2.2 × 10^8^ to about 5.1 × 10^7^ cells/mL) was not at all altered by the presence of either CuO-NPs at MBC (from 2.2 × 10^8^ to about 5.7 × 10^7^ cells/mL) or CuO-NPs pre-incubated with EDA (from 2.2 × 10^8^ to about 4.3 × 10^7^ cells/mL) (Table 3). This result signified that the free amine groups of EDA readily interacted with CuO-NPs, modifying the NPs to less-toxic form and making the concentration of the modified particles (6 µg/mL) to be non-toxic.

**Table 3:** Effect of EDTA and EDA on the viability of CuO-NP treated *E. coli K-12* cells in saline. The data represent mean values ± S.D. (standard deviation) from three independent experiments.

Samples	Number of viable cells
0.0 µg/ml	2.2 ± 0.48 × 10^8^
6.0 µg/ml CuO-NP	2.8 ± 0.37 × 10^5^
6.0 µg/ml CuO-NP + 2 mM EDTA	2.5 ± 0.24 × 10^5^
6.0 µg/ml CuO-NP + 2 mM EDTA pre-incubated for 30 min	2.6 ± 0.31 × 10^5^
2 mM EDTA	4.8 ± 0.51 × 10^7^
6.0 µg/ml CuO-NP + 2 mM EDA	5.7 ± 0.28 × 10^7^
6.0 µg/ml CuO-NP + 2 mM EDA pre-incubated for 30 min	4.3 ± 0.36 × 10^7^
2 mM EDA	5.1 ± 0.43 × 10^7^

**Figure 6:**
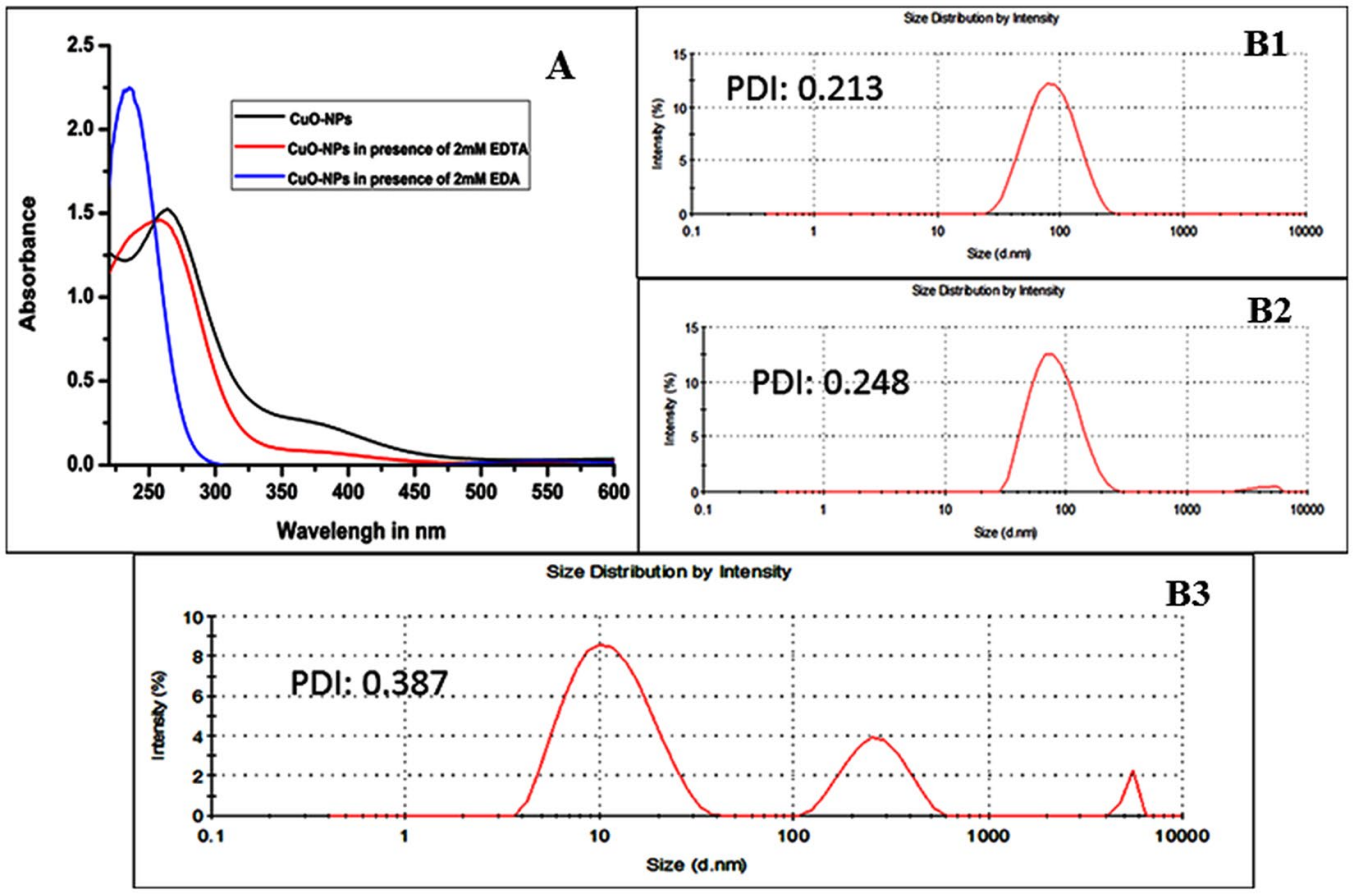
The absorption spectrum (**A**) and hydrodynamic size distribution (**B**) of CuO-NP in saline in the presence of EDTA and EDA. B1: CuO-NP alone, B2: CuO-NP and 2 mM EDTA and B3: CuO-NP and 2 mM EDA.

The investigation was further carried out to understand whether the modification of CuO-NP by EDA was similar to the modification by amino acids with free amino groups. For this, a spectrophotometric study was performed. The NP itself had two absorption peaks – one sharp peak at 266 nm and one wide peak at 370 nm; by the presence of EDA (2 mM), the peak at 266 nm was blue-shifted to 235 nm with an increase of peak intensity and that at 370 nm was completely abolished (Fig. 6A). Similar changes in spectral pattern of CuO-NP in presence of amino acid mixture i.e., shifting of the 266 nm peak to 230 nm and the abolition of the peak at 370 nm was reported earlier^20^. On the other hand, such drastic changes in the spectral pattern of CuO-NP did not occur by the presence of EDTA (2 mM); here, the absorption peak of the NP at 266 nm was slightly shifted to 256 nm and the peak at 370 nm did not disappear but its intensity decreased (Fig. 6A).

The alteration happened to CuO-NPs by EDA, but not by EDTA, was further monitored by determining the size of the NP, suspended in saline in presence of EDA and EDTA separately. The hydrodynamic size of CuO-NPs (83.5 nm, Fig. 6B1), as determined by the particle size analyzer (Malvern, Nano-ZS), remained almost unchanged (82 nm, Fig. 6B2) in presence of EDTA (2 mM). This implied that no modification of CuO-NPs by EDTA had occurred through any complex formation. On contrary, the interaction of CuO-NPs with EDA (2 mM) generated three types of particle population; majority particles (about 73%) were around 15 nm in size, 24% of the particles were about 274 nm in size and few particles (about 3%) were very large in µm range (Fig. 6B3). We reported earlier^20^ that a similar pattern of the result was obtained when CuO-NPs were incubated with amino acid lysine; two types of particle were generated – 70% particles were of size 20 nm and 30% particles were of size 230 nm. It was proposed there^20^ that the particles of size 20 nm were made of the PVA (the stabilizing agent of CuO-NP) that were displaced from the NP by the alternative stabilizer lysine and the particles of size 230 nm were CuO-NP-lysine complex or in other words, CuO-NPs were stabilized by lysine. Here also the same phenomenon was likely to be happened due to interaction between the NP and EDA; 15 nm sized particles were made of the released PVA from the NP, 274 nm sized particles were CuO-NP-EDA complex and ‘µm’ ranged particles were perhaps few aggregates of CuO-NP-EDA complex. It should be mentioned here that the low value of PDI (poly dispersive index) up to 0.3 represents good quality (homogeneous) suspension of particles, of which most were equal in size and therefore, the values of PDI, shown in Fig 6B1–B3, indicated that most of the particles of any population were homogeneously dispersed and of equal size, as measured. In short, the above results can be summarized as – EDA, added in CuO-NPs suspension in saline had bound, modified and stabilized the NPs by its free amine group, perhaps with simultaneous release of the original stabilizer PVA from the NPs and thereby made the modified NPs less potent cell killer than the unmodified ones; moreover, no effect on the cell-killing efficacy of the NP in presence of the ion chelating agent EDTA demonstrated clearly that the bacterial cell killing in saline by CuO-NP was caused not by any Cu-ions leached out from the NPs.

### Antibacterial activity of CuCl_2_ and CuO-NPs on *E. coli W31101ΔCF* cell in LB medium

It was known that the copper ions from the external media entered into bacterial cells through outer membrane porins OmpC and OmpF^24,25^. Therefore, to establish once more the ‘particle-specific effect’ rather than ‘ion-specific one’ behind the bactericidal potency of CuO-NPs, the experiment was carried out on *E. coli* strain W31101ΔCF, having mutations in both *ompC* and *ompF* genes. The mutant strain could not transport external copper ions inside the cells^25^; the cells were found to survive under the certain concentration of the copper ion in the external environment, above which the cells could not survive due to osmotic shock^26,27^. In our case, the mutant W31101ΔCF cells survived in LB nutrient medium without any growth and cell division (bacterio-static) by the presence of Cu-ions equivalent to 90 µg/mL of CuCl_2_ and thus this concentration was the minimum inhibitory concentration (MIC, defined as the concentration of an antibacterial agent in the growth medium that causes complete inhibition of bacterial growth without cell killing even after overnight incubation^20^) of CuCl_2_ to the cells. The MBC of CuCl_2_ to the cells was determined to be 110 µg/mL (Table 4). On the other hand, the MIC and MBC of CuO-NP for the W31101ΔCF cells in LB medium were determined to be about 60 and 75 µg/mL respectively (Table 4). As CuO-NPs could kill the mutant W31101ΔCF cells at concentration (above 60 µg/mL) lower than its ionic tolerance limit (about 90 µg/mL) to the cells, this result further elucidated that the mode of antibacterial action of CuO-NP was not ‘ion-specific’ effect, rather it was a ‘particle specific’ one.

**Table 4:** Number of viable *E. coli W31101ΔCF* cells after 18 h incubation in LB medium with different concentrations of CuCl_2_/CuO-NP. Results are representatives of three independent experiments. Data represent mean values ± S.D.

Concentration of CuO-NP	Number of viable cells	Concentration of CuCl_2_	Number of viable cells
0.0 µg/mL	2.9 ± 0.37 × 10^6^	0.0 µg/mL	2.9 ± 0.37 × 10^6^
60 µg/mL (MIC)	3.1 ± 0.45 × 10^6^	90 µg/mL (MIC)	4.2 ± 0.61 × 10^6^
75 µg/mL (MBC)	6.5 ± 0.28 × 10^3^	100 µg/mL (MBC)	1.7 ± 0.53 × 10^3^

## Conclusion

Earlier we reported that in a nutrient medium, CuO-NPswere modified by medium organics with a considerable increase in the size of the NPs^20^. Therefore, the reported antibacterial efficacy of CuO-NP was not of the original particles, but of the modified ones. Here we report that the original unmodified CuO-NPs was much more bacteriotoxic than the modified particles. Our experimental findings demonstrate that when bacteria (Gram negative or positive) were suspended in organics-free saline (0.9% NaCl) and were treated with CuO-NP, the unmodified particles had MBC value in the range of 6–7.5 µg/mL, which was much less than the range of 160–195 µg/mL of the modified NPs in organics-full nutrient medium. Here we also find that the unmodified CuO-NPs, like modified ones^20^, induced ROS in bacterial cells and this ROS triggered cellular lipid peroxidation, protein oxidation, and DNA degradation. Since cellular ROS generated by modified particles triggered lipid peroxidation and DNA degradation only^20^, therefore, the additional protein oxidation in cells, treated with the unmodified NPs, perhaps played a crucial role behind their high bactericidal activity, compared to the modified CuO-NP. It also came out from this study that the MBC value of the unmodified CuO-NP (6 µg/mL) for *E. coli* did not alter with addition of metal ion chelator EDTA; this result signified that the chemical mode of bactericidal action of CuO-NP was not an ion-mediated effect, but an effect of the particle itself as a whole.

From the application aspect, our CuO-NP with high antibacterial activity in unmodified form can be used as a potent surface sterilizer. Moreover, recent reports on bio-conjugation of antibiotics with nanoparticles, especially for Gold and silver, depicted remarkable success in the enhancement of the antibacterial activity of the clinically approved drugs^28,29^. Therefore, in future, venture may be taken on the bio-conjugation of drugs with our CuO-NP to develop more potent drugs, perhaps through synergistic action of drug and CuO-NP. Presently, work is being planned in our laboratory to encapsulate/entrap our CuO-NP within calcium phosphate NP to develop effective nano-fertilizer.

## Materials and Methods

### Assays of antibacterial action of CuO-NP

#### Determination of the MBC of CuO-NP

MBC of an antibacterial substance is defined as the concentration, the presence of which in growth medium results in 99.9% cell killing on overnight (18 h) incubation^9,10^. To determine the MBC of CuO-NP, the experiment was done as follows: a drop of about 1.0 µL of 20% glycerol-preserved culture of *E coli K-12* cells was taken in 5 mL Luria-Bertani (LB) medium^20^, mixed well and was finally allowed to grow overnight (14–16 h) at 37 °C incubator with mild shaking. On the next day, the overnight grew culture was inoculated at a concentration of 1% v/v in fresh LB medium and was further grown freshly in a 37 °C shaking incubator up to log phase [(O.D) 600 nm ≈ 0.3 i.e., the cell number of about 10^8^ cells/mL]. The grown cells were then centrifuged at 8000 rpm for 5 min. The cell pellet was suspended in the same volume of starvation buffer (SB)^20^, followed by incubation at 37 °C for 1 h with shaking, so that the cells were exhausted of ATP and every cell came to the same state of growth. Such synchronized cells were washed and finally suspended in saline (0.9% NaCl). The saline suspended bacterial cells were treated with CuO-NP at varying concentrations, followed by incubation with shaking for 18 h. Cell numbers per mL of the cultures before and after incubation were determined by the technique of colony counts over agar plates, as described in^9^.

#### Determination of change in cellular membrane potential by CuO-NP treatment

Bacterial cell death causes decay of cell membrane potential^22^. This potential was determined by flow Cytometric method, labeling the cells by the dye, 3,3′-diphenylthiocarbocyanine iodide^13^. Principle of the method is that the dye gets incorporated into the cell membrane in a potential-dependent way and in the hydrophobic environment of the membrane the non-fluorescent dye becomes fluorescent; therefore, any decrease in membrane potential causes a release of some dye from the membrane, finally quenching the fluorescence intensity. Thus any alteration of cell membrane potential can be measured from the change in fluorescence intensity of the bound dye. To measure membrane potential, grown *E. coli K12* cells were first synchronized as described above and the synchronized cells were transferred through centrifugation in saline, followed by incubation with CuO-NP of concentration equal to MBC for 18 h. The treated cells were diluted 100-folds (to about 10^6^ cells/mL) and were taken in a flow-cytometer tube to stain with 10 µL of 3 mM dye (3,3′-diphenylthiocarbocyanine iodide) for 2 h. Fluorescence was then collected in the red channel (FL2; 575 nm) of the flow cytometer (FACS Calibur, Becton and Dickinson) equipped with an argon laser (488 nm) and standard filter set-up. All the data were analyzed using ‘Cell Quest-Pro’ software (Becton and Dickinson) and membrane potential was qualitatively compared using dot plot analysis.

### Analysis of oxidative stress-induced damages in CuO-NPtreated cells

#### Estimation of ROS production in CuO-NPtreated cells

The extent of ROS production in bacterial cells was estimated by using a membrane-permeable dye 2′,7′-dichlorodihydrofluorescein diacetate (DCFH_2_-DA). Principle of the assay is that during passage through the cell membrane, cellular esterase hydrolyzes DCFH_2_-DA to DCFH_2_, which is oxidized by ROS to highly fluorescent DCF^13,20^. So, the extent of DCF production estimates ROS production in cells. Therefore, to estimate cellular ROS, synchronized cells were treated with CuO-NP at different concentrations in saline for 18 h, washed through centrifugation and finally suspended in saline at concentration of ~2 × 10^6^ cells/mL. A volume of 10 µM DCFH_2_-DA was added to the suspended cells, which were then incubated in dark for 2 h prior to measurement of ROS, using flow cytometer.

#### Estimation of lipid peroxidation (LPO) in CuO-NP-treated cells

The extent of LPO in the NP-treated cells was estimated by thiobarbituric acid (TBA) assay method. Principle of the assay is that LPO or in other words, oxidation of polyunsaturated fatty acids produces malondialdehyde (MDA), which is known to react with TBA producing a chromophoric adduct of absorption maxima at 532 nm^13,20^. So, the extent of absorption intensity at 532 nm estimates LPO in cells. Here, synchronized cells, treated with NP for 18 h, were washed with and suspended in fresh saline. Then 1.0 mL of cell suspension was lysed by adding 100 µL SDBME buffer^30^. Lysed cell extract (100 µL) was mixed with 200 µL of TBA-TCA (tri-chloroacetic acid)-HCl reagent [15% TCA and 0.375% TBA dissolved in 0.25 (N) HCl] and heated for 15 min in boiling water bath. After cooling, flocculent precipitates were removed by centrifugation at 12,000 rpm for 10 min. The absorbance of the supernatant was measured at 532 nm and the level of lipid peroxidation was determined according to the following equation^31^,

$$\begin{array}{ccc}{\rm{L}}{\rm{P}}{\rm{O}}\,{\rm{a}}{\rm{c}}{\rm{t}}{\rm{i}}{\rm{v}}{\rm{i}}{\rm{t}}{\rm{y}} & = & (({\rm{A}}{\rm{b}}{\rm{s}})532\times {\rm{T}}{\rm{o}}{\rm{t}}{\rm{a}}{\rm{l}}\,{\rm{r}}{\rm{e}}{\rm{a}}{\rm{c}}{\rm{t}}{\rm{i}}{\rm{o}}{\rm{n}}\,{\rm{v}}{\rm{o}}{\rm{l}}{\rm{u}}{\rm{m}}{\rm{e}})/({\rm{S}}{\rm{a}}{\rm{m}}{\rm{p}}{\rm{l}}{\rm{e}}\,{\rm{v}}{\rm{o}}{\rm{l}}{\rm{u}}{\rm{m}}{\rm{e}}\\  &  & \times {\rm{M}}{\rm{o}}{\rm{l}}{\rm{a}}{\rm{r}}\,{\rm{e}}{\rm{x}}{\rm{t}}{\rm{i}}{\rm{n}}{\rm{c}}{\rm{t}}{\rm{i}}{\rm{o}}{\rm{n}}\,{\rm{c}}{\rm{o}}-{\rm{e}}{\rm{f}}{\rm{f}}{\rm{i}}{\rm{c}}{\rm{i}}{\rm{e}}{\rm{n}}{\rm{t}}\,(\in =1.56\times {[10]}^{(5)}{[{\rm{m}}{\rm{o}}{\rm{l}}]}^{(-1)}{[{\rm{c}}{\rm{m}}]}^{(-1)})\\  &  & \times {\rm{P}}{\rm{r}}{\rm{o}}{\rm{t}}{\rm{e}}{\rm{i}}{\rm{n}}\,{\rm{c}}{\rm{o}}{\rm{n}}{\rm{c}}{\rm{e}}{\rm{n}}{\rm{t}}{\rm{r}}{\rm{a}}{\rm{t}}{\rm{i}}{\rm{o}}{\rm{n}})\,{\rm{n}}{\rm{M}}/{\rm{m}}{\rm{g}}/{\rm{u}}{\rm{n}}{\rm{i}}{\rm{t}},\end{array}$$

where protein concentration in the cell lysate was measured by Brad-Ford method^32^.

### Estimation of protein oxidation in CuO-NP treated cells

The extent of protein oxidation in the NP-treated cells was measured by the method described in^13^. Principle of the method is that cellular ROS oxidizes proteins to protein-carbonyls and these carbonyl compounds readily react with di-nitro-phenyl-hydrazine to form a stable 2,4-dinitrophenol (DNP) of absorption maxima at 370 nm. Thus, the measure of absorbance intensity of DNP at 370 nm directly reflects the level of protein oxidation in cells. Here, the lysate of NP-treated cells, prepared as described in the previous subsection, was centrifuged at 12000 rpm for 10 min at 4 °C and the supernatant was collected. 20% ice-cold TCA was added to the supernatant and left at (−) 20 °C for about 1 h until white precipitates appeared. A sticky pellet of the precipitates was collected by further centrifugation at 12,000 rpm for 15 min at 4 °C. The pellet was incubated with 0.4 mL of 0.2% 2,4-dinitrophenylhydrazine (DNPH, made in 2 N HCl) in dark for 1 h, with vortexing at intervals of 10 min. The hydrazone derivative of proteins was first extracted by simple precipitation with 10% ice-cold TCA; the precipitate was then suspended in ethanol-ethyl acetate (1:1 v/v) and re-extracted with 10% cold TCA. Finally, the collected precipitate was dissolved in 6 M guanidine hydrochloride (Gu-HCl), kept at 37 °C for 15 min, centrifuged at 5000 rpm for 2 min(to get rid of the un-dissolved residues) and the supernatant was collected to measure its absorbance at 370 nm.

### Estimation of genomic DNA degradation in CuO-NPtreated cells

After 18 h treatment of synchronized cells with CuO-NP, 3.0 mL treated culture of cell concentration (10^7^ cells/mL) was centrifuged at 8000 rpm. The resulting cell pellet was suspended in 600 µL lysis buffer (10 mL lysis buffer was composed of 9.34 mL TE buffer +600 µL of 10% SDS + 60 µL of proteinase-K of 20 mg/mL stock) and kept at 37 °C for 1 h for complete lysis. The lysate was then subjected to phenol-chloroform extraction to remove proteins; genomic DNA in the extract was isolated by cold ethanol precipitation^33^. Isolated DNA was finally electrophoresed in 1% agarose gel^34^.

## Supplementary information

Surface Modification by Media Organics Reduces the Bacterio-toxicity of Cupric Oxide Nanoparticle against Escherichia coli

## Data Availability

All data generated or analyzed during this study are included in this published article (and its Supplementary Information files).
