# Supplementary material for: Surface Modification by Media Organics Reduces the Bacterio-toxicity of Cupric Oxide Nanoparticle against Escherichia coli
Source: Sci Rep. 2019 Oct 25;9:15364. doi: 10.1038/s41598-019-51906-2 (PMC6814817; doi:10.1038/s41598-019-51906-2)

Supplementary information

Surface Modification by Media Organics Reduces the Bacterio-toxicity of Cupric Oxide Nanoparticle against *Escherichia coli*

Ruchira Chakraborty and Tarakdas Basu†

Department of Biochemistry and Biophysics, University of Kalyani, Kalyani – 741235, West Bengal, India

† Corresponding author:tarakdb@yahoo.com

Table S1: Numbers of viable *E. coli K-12* cells in saline after treatment with different concentrations of CuO-NPs for different time intervals. The data represent mean values ± S.D. (standard deviation) from three independent experiments.

**Concentration of CuO-NP**	**Number of viable cells after treatment with CuO-NP for**				
	**1h**	**3h**	**6h**	**12h**	**18h**
0.0µg/ml	2.6 ± 0.51 × 10^8	2.55 ± 0.43 × 10^8	2.3 ± 0.26 × 10^8	2.12 ± 0.32 × 10^8	2.0 ± 0.15 × 10^8
1.5µg/ml	2.5 ± 0.37 ×10^8	2.21 ± 0.28 × 10^8	7.92 ± 0.43 × 10^7	6.7 ± 0.37 × 10^7	6.3 ± 0.19 × 10^7
3.0µg/ml	2.52 ± 0.48 × 10^8	1.16 ± 0.63 × 10^8	4.89 ± 0.31 × 10^7	1.78 ± 0.23 × 10^7	1.2 ± 0.38 × 10^7
4.5µg/ml	2.4 ± 0.59 × 10^8	8.98 ± 0.38 × 10^7	7.31 ± 0.24 × 10^6	5.98 ± 0.47 × 10^6	5.7 ± 0.23 × 10^6
**6.0µg/ml**	2.35 ± 0.63 × 10^8	7.72 ± 0.49 × 10^6	**5.35 ± 0.58 × 10^5**	**4.21 ± 0.35 × 10^5**	**3.1 ± 0.16 × 10^5**
7.5 µg/ml	2.1 ± 0.52 × 10^8	1.76 ± 0.48 × 10^5	3.29 ± 0.27 × 10^4	1.98 ± 0.61 × 10^3	1.6 ± 0.41 × 10^2
9.0 µg/ml	7.25 ± 0.49 × 10^6	1.6 ± 0.5 × 10^2	0× 10	0× 10	0×10

Table S2: Number of viable *S. aureus* cells after 18h of incubation in saline with varying concentrations of CuO-NP / lysine-modified CuO-NP. Results are representatives of three independent experiments. Data represent mean values ± S.D.

**Concentration of CuO-NP**	**Number of viable *S. aureus* cells**
0.0μg/ml	$3.1 \pm 0.25 \times 10^{8}$
3.0μg/ml	$7.8 \pm 0.58 \times 10^{7}$
4.5 μg/ml	$1.2 \pm 0.45 \times 10^{7}$
6.0μg/ml	$3.9 \pm 0.28 \times 10^{6}$
**7.5 μg/ml**	**$4.6 \pm 0.23 \times 10^{5}$**
9.0 μg/ml	$6.5 \pm 0.29 \times 10^{3}$
12.0 μg/ml	0×10

Table S3: Number of viable *E. coli K-12* cells in saline after treatment with different concentrations of EDTA and EDA for 18h. The data represent mean values ± S.D. (standard deviation) from three independent experiments.

**Concentration of EDTA/ EDA**	**Number of viable cells**	
	**EDTA**	**EDA**
0.0mM	$2.8 \pm 0.41 \times 10^{8}$	$2.8 \pm 0.41 \times 10^{8}$
1.0mM	$5.7 \pm 0.34 \times 10^{7}$	$6.2 \pm 0.28 \times 10^{7}$
2.0mM	$4.8 \pm 0.51 \times 10^{7}$	$5.1 \pm 0.43 \times 10^{7}$
4.0mM	$1.8 \pm 0.28 \times 10^{5}$	$2.1 \pm 0.30 \times 10^{5}$
5.0mM	$7.6 \pm 0.31 \times 10^{4}$	$6.8 \pm 0.46 \times 10^{4}$
10mM	$1.5 \pm 0.17 \times 10^{2}$	$1.3 \pm 0.21 \times 10^{2}$

Figure S1: Changes in absorption spectrum of CuO-NP suspension (360µg/mL) with stepwise addition of amino acids A) lysine, B) Glutamate, C) serine and D) glycine, at increasing concentrations (0, 10, 25, 50, 75, 100, 200, 300 and 500 µg/mL).

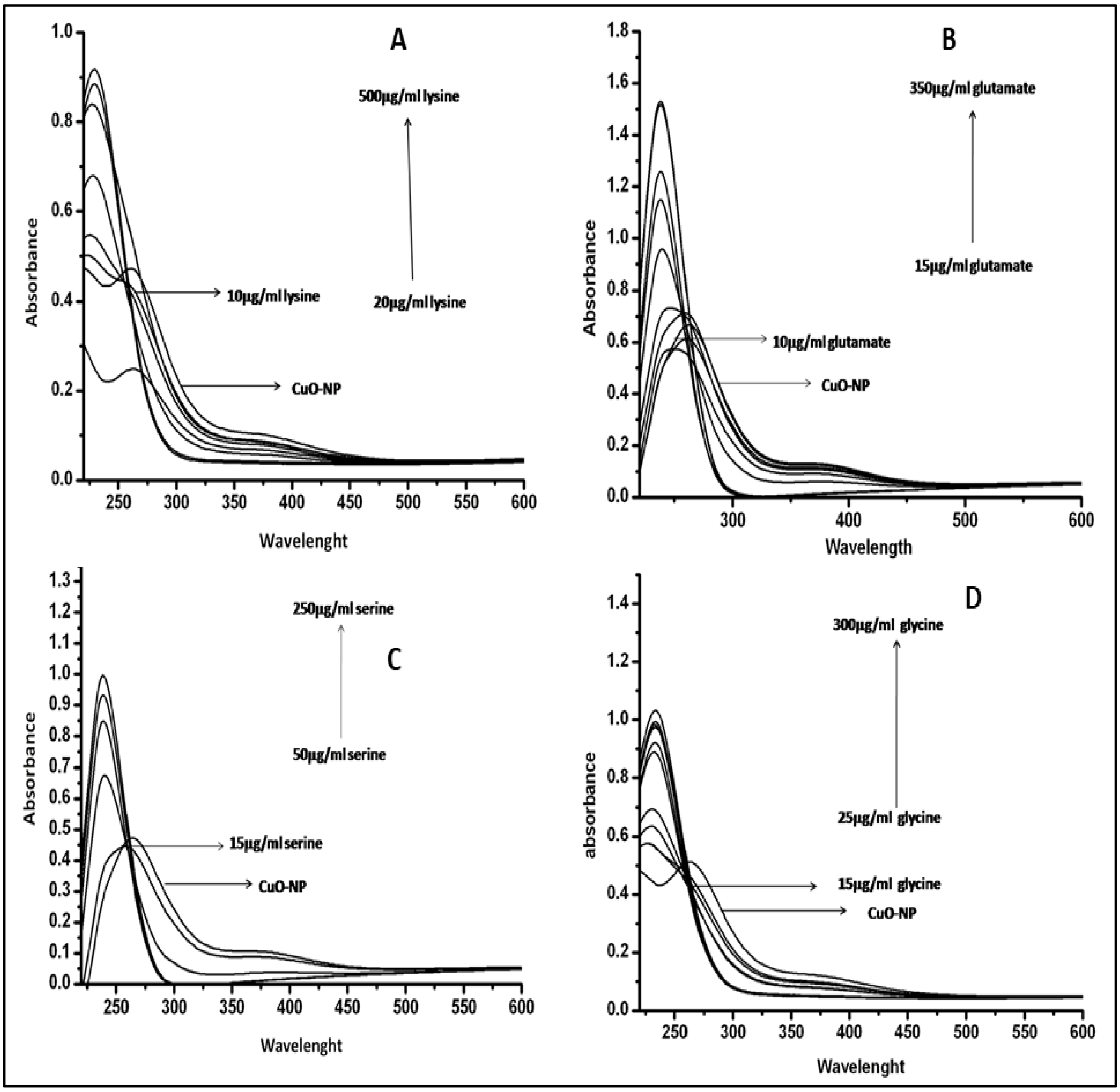

Figure S2: Spectrophotometric analysis of stepwise addition of lysine-modified CuO-NP in LB.

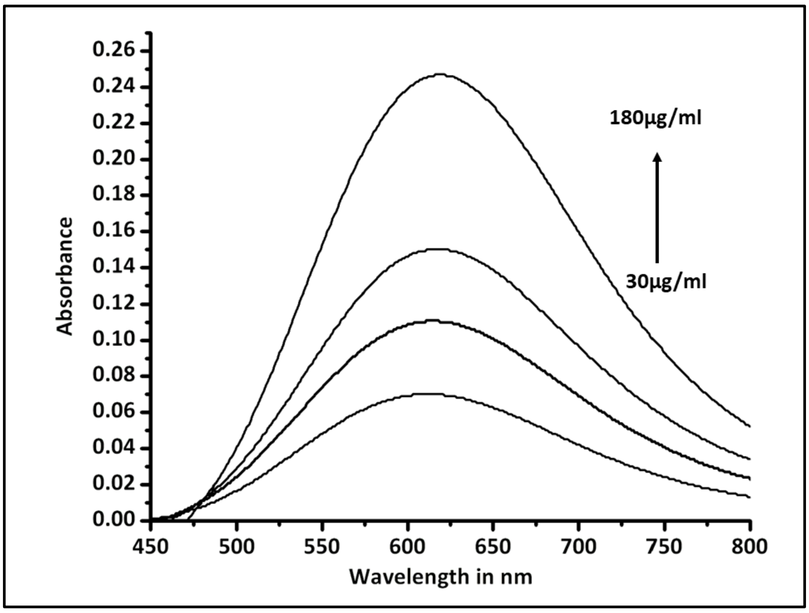

Supplement: Supplementary file 1 — Surface Modification by Media Organics Reduces the Bacterio-toxicity of Cupric Oxide Nanoparticle against Escherichia coli [file 41598_2019_51906_MOESM1_ESM.pdf]
